# IGF-1 Receptor and Adhesion Signaling: An Important Axis in Determining Cancer Cell Phenotype and Therapy Resistance

**DOI:** 10.3389/fendo.2015.00106

**Published:** 2015-07-03

**Authors:** Orla T. Cox, Sandra O’Shea, Emilie Tresse, Milan Bustamante-Garrido, Ravi Kiran-Deevi, Rosemary O’Connor

**Affiliations:** ^1^Cell Biology Laboratory, BioSciences Institute, School of Biochemistry and Cell Biology, University College Cork, Cork, Ireland

**Keywords:** IGF-1R, PDLIM2, adhesion, EMT, signaling, phenotype, resistance

## Abstract

IGF-1R expression and activation levels generally cannot be correlated in cancer cells, suggesting that cellular proteins may modulate IGF-1R activity. Strong candidates for such modulation are found in cell-matrix and cell–cell adhesion signaling complexes. Activated IGF-1R is present at focal adhesions, where it can stabilize β1 integrin and participate in signaling complexes that promote invasiveness associated with epithelial mesenchymal transition (EMT) and resistance to therapy. Whether IGF-1R contributes to EMT or to non-invasive tumor growth may be strongly influenced by the degree of extracellular matrix engagement and the presence or absence of key proteins in IGF-1R-cell adhesion complexes. One such protein is PDLIM2, which promotes both cell polarization and EMT by regulating the stability of transcription factors including NFκB, STATs, and beta catenin. PDLIM2 exhibits tumor suppressor activity, but is also highly expressed in certain invasive cancers. It is likely that distinct adhesion complex proteins modulate IGF-1R signaling during cancer progression or adaptive responses to therapy. Thus, identifying the key modulators will be important for developing effective therapeutic strategies and predictive biomarkers.

## Introduction: IGF-1R Signaling in Cell–Matrix Adhesion Complexes and Cell Migration

**Figure 1:**
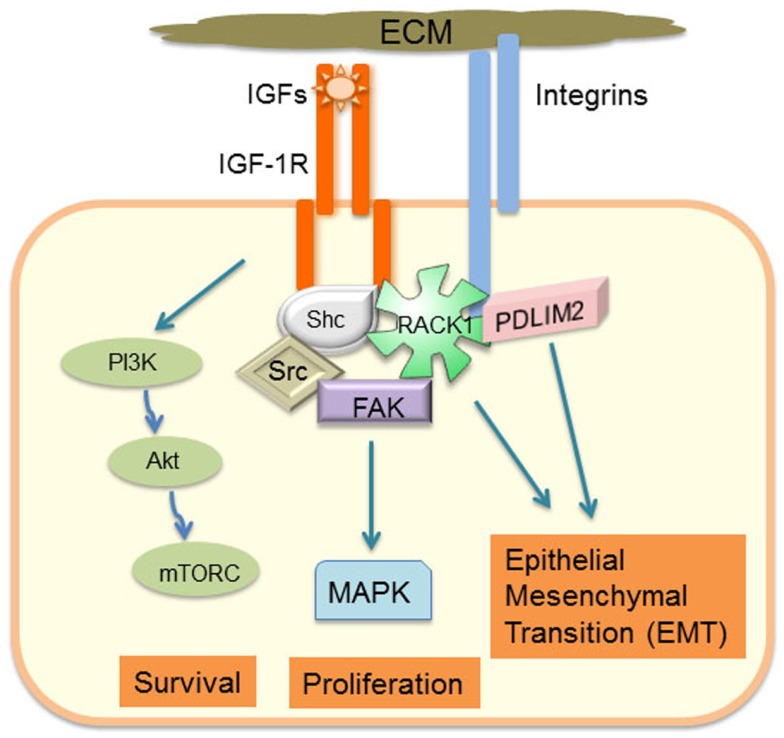
**Schematic model of how adhesion-regulated IGF-1R signaling has a critical role in determining cancer cell phenotype**.

Dynamic cooperative signaling interactions between the IGF-1R and integrins are necessary for the growth and migration of normal cells and also for invasiveness and metastasis of cancer cells. Examples include how in normal cells, IGF-1R expression and activation are required for fibroblast migration, integrating signals from the extracellular matrix (ECM) via β1 integrin and RACK1 scaffolding protein (1–4) (summarized in Figure 1). In vascular smooth muscle cells, IGF-1 stimulated cell migration and division requires αvβ3 integrin cooperation [reviewed in Ref. (5)]. α5β1 integrin signaling is strongly interactive with IGF-1R signaling in prostate cancer cells, and IGF-1R has been shown both to associate with and enhance the stability of β1 integrin (6). Furthermore, the IGF-1R interaction with αv integrin in both normal and colon cancer cells is disrupted upon IGF-1 stimulation, which correlates with increased cell migration (7).

In addition to its well-described role in cooperative signaling with integrins to promote normal cell growth and migration [reviewed in Ref. (8, 9)], IGF-1R and integrin collaborative signaling in cancer cells is implicated with an epithelial mesenchymal transition (EMT) phenotype (10–12). IGF-1R can promote integrin stability, and in prostate tumor models, integrin expression is required for growth *in vivo* and proliferation *in vitro* (6, 13). Alternations and co-expression of IGF-1R and adhesion signaling components have been reported in several different cancers. For example, a recent analysis of IGF-1 and ECM-induced signaling components in metastatic breast tumors demonstrated that compared with normal or primary cancer tissues, β1 integrin and fibronectin are more clearly co-located at the leading edge of tumors, which also correlates with active Akt and Erks (14).

Integrin engagement leads to formation of transient nascent adhesions that can mature into focal adhesions tethered to actin stress fibers. The assembly and disassembly of these focal adhesions is necessary for cell migration and involves coordinated activation of focal adhesion kinase (FAK), Src, and Rho GTPases (15). Activated IGF-1R can be recruited into a signaling complex with β1 integrin via scaffolding with RACK1 and FAK (1–4, 6, 16, 17) (Figure 1). RACK1 acts as a scaffold to facilitate activation of FAK, and IGF-1 stimulates dephosphorylation of FAK, which is associated with dissolution of focal adhesions (2, 18, 19). This complex contains key IGF-1-responsive signaling components, including IRS-1, IRS-2, Shc, Src, PP2A, Shp-2, and c-Abl, which have a particular role in regulating activity of the Erk signaling cascade. Indeed, cell adhesion or integrin ligation is required for optimal IGF-1-mediated activation of Shc and Erks (5, 20, 21). The intensity and duration of Erk phosphorylation in response to IGF-1 in the presence of cell adhesion may be a key determinant of cell phenotype and, in particular, EMT potential of cancer cells. For example, RACK1 over-expression biases the IGF signaling response toward increased Erk phosphorylation with concomitant increases in IGF-1 mediated cell proliferation and migration (1). G-protein coupled receptor (GPCR) engagement is also a component of adhesion-regulated IGF signaling [reviewed in Ref. (22)], which biases IGF-1-mediated Erk activation (23). Several extracellular matrix and intracellular proteins have the potential to influence the level of IGF-1R association with adhesion receptors and subsequent signaling. These include ECM components, such as fibronectin and collagen; proteoglycans, such as decorin, which regulates IGF-1R activation levels and internalization (24); and IGF binding proteins (IGFBPs) that can associate with ECM proteins and modulate IGF ligand activity or adhesion signaling (25, 26). Recent studies have also implicated the discoid domain receptor 1 (DDR1), which is a receptor tyrosine kinase (RTK) that becomes activated by collagen ligation (27, 28), and may be important for cell matrix adhesion and growth regulation. DDR1 is also over-expressed in many cancers (28) and it associates with the IGF-1R (29). DDR1 association with the IGF-1R regulates IGF-1R trafficking and expression levels and promotes collagen-dependent and -independent phosphorylation of DDR1. Hence, DDR1 is a newly characterized adhesion receptor that regulates IGF-1R expression and signaling in cancer cells (29). Interestingly, cancer genome sequencing studies indicate that head and neck cancers have many alterations in both IGF-1R and the DDR2 collagen receptor (http://cancergenome.nih.gov). Thus, IGF-1R levels alone will not necessarily determine cancer cell responses to IGF-1 and anti-IGF-1R therapies, as IGF-1R activity and downstream signaling are influenced by adhesion signals and activation of other signaling pathways, which are discussed further below.

## IGF-1R Regulation of Cell–Cell Adhesion Complexes

IGF-1R can be found associated with E-cadherin in cell–cell adhesion complexes of normal corneal epithelial cells (30) and in several cancer cell types (7, 31–34). IGF-1 can stimulate cell–cell adhesion associated with survival and reduced migration in both 2D and 3D models (31, 32, 34). Indeed, in MCF-7 breast cancer cells, IGF-1R interacts with, and regulates expression of the scaffolding protein zonula occludens protein 1 (ZO-1) at E-cadherin complexes, thereby enhancing the E-cadherin-mediated cell–cell adhesion (32, 34). However, IGF-1R activation can also promote cell migration in both normal and cancer cells [reviewed in Ref. (9, 35, 36)]. Whether IGF-1R promotes cell adhesion or disruption of the E-cadherin adhesion complexes appears to be cell-type specific. For example, it has been shown that the interaction of active IGF-1R and E-cadherin is required for normal murine blastocyst formation (37). However, activation of the IGF-1R by either IGF-1 (38) or IGF-II (33) has been also shown to disrupt cell–cell contacts with concomitant redistribution of E-Cadherin and beta catenin from cell adhesion complexes to the cytoplasm or cytoplasm and nucleus, respectively, which is permissive to EMT and cell migration (7, 33). Integrin engagement by ECM is associated with dissolution of cell–cell junctions, and the integrin-activated signaling pathway to FAK, Src, and small GTPases (including RhoA), and Rho Kinase can promote phosphorylation of adherence junction proteins or the stability of E-cadherin expression (15). There is also evidence for regulation of integrin adhesion by adherens junctions (AJ), where a key signaling intermediary is thought to be the Ras family GTPase Ras-related protein 1 (Rap1), which becomes activated upon AJ disassembly and is associated with focal adhesion formation (39). Interestingly, activated IGF-IR also transiently activates Rap1 and recruits it to sites of cell motile protrusion, whereas Rap1 remains in site of cell–cell contact in the absence of IGF-1R activation (40). IGF-1R and Rap1 expressions were both reported to exhibit increased expression in invasive breast cancer (41), again suggesting that IGF-1R contributes to the invasive switch in cancer.

## IGF-1R Signaling in Cancer Phenotype, EMT, and Adaptive Responses to Therapy

Adhesion signaling in cooperation with IGF-1R may have an important role in the responses of cells to kinase inhibitors, chemotherapeutic agents, and other therapies. This adaptive response may be related to an EMT phenotype, whereby cells acquire a mesenchymal phenotype allowing them to invade and migrate and has also been likened to a stem-like phenotype. IGF-1 can induce transcription of drivers of EMT including the E-cadherin transcriptional regulators, Snail and Zeb (33, 42–45). In ovarian cell models, mechanisms of adaptive resistance to PI3-K/mTOR inhibitors were attributed to extracellular matrix attachment accompanied by up-regulation of IGF-1R and other pro-survival proteins (46). Recent reports on IGF-1R and Her2 cooperation in invasiveness also indicate that the major biological effect facilitated by IGF-1R is invasion mediated by Src and FAK (47). The authors suggest that this effect is very likely to depend on integrin signaling, which would indeed be consistent with the published studies on Src, FAK, and integrin action in IGF-1R signaling.

A dataset for IGF-1-mediated activation of Akt and Erks in 50 breast cancer cell lines is available in the library of integrated network-based cellular signatures (LINCS) consortium website from a study by Niepel et al., which collated the responses to ligand stimulation and tyrosine kinase inhibitors for a panel of growth factors (48, 49). It is clear from this dataset that IGF-1R expression levels vary substantially across the 50 cell lines and that autophosphorylation on Y1131 (activity) of the IGF-1R does not necessarily correlate with receptor levels or downstream signaling events. This implies regulation or biasing of IGF-1R activity and signaling output by other signaling pathways, as discussed in the previous section. These pathways may include those activated by fibroblast growth factor (FGF), which biases toward Erk signaling in this study (48), epidermal growth factor (EGF), c-Met, or the Wnt signaling pathway (50–53). It is also becoming increasingly clear that expression and activation of these pathways may have important implications for responses to anti-IGF-1R therapy, as has been shown for DVL3 signaling (54).

In addition to its role in stem cell renewal, the Wnt pathway is increasingly recognized as an important driver of EMT, although the mechanisms and interactions with RTK and adhesion signaling are not yet well understood [reviewed in Ref. (52)]. Wnt signaling acts through 10 known seven-transmembrane Frizzled (Fzd) family receptors and three Disheveled (DVL) isoforms (55, 56), which can activate either a canonical signaling pathway through beta catenin; a non-canonical pathway that includes Rho, Rac, PKCs, Jnk, and other proteins; or the alternative Wnt 5/Fzd2 pathway that is mediated by STAT3 and Fyn kinase (57). IGF-1R signaling can intersect with the canonical Wnt pathway at the level of GSK3 beta phosphorylation and inactivation, leading to stabilization and transcriptional activation of beta catenin. IGF-1R inhibition also modifies Wnt pathway activity (58), and Wnt pathway components may modulate IGF-1R signaling (54). DVL3, a component of Wnt signaling pathways, was recently identified as a modifier of response to IGF-1R antibody or tyrosine kinase inhibitors, and DVL3 expression can alter the kinetics of IGF-1-mediated Erk activity (54). There is also evidence for altered levels of these Wnt pathway proteins in different stages of cancer differentiation and regulation of canonical and non-canonical Wnt signaling during cancer progression (59–61).

## Adhesion Signaling Integrated with Control of Gene Expression: Role for PDLIM2

**Figure 2:**
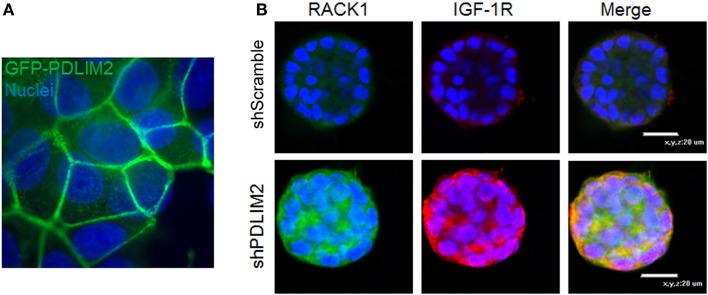
**(A)** PDLIM2 is expressed at cell–cell adhesions: MCF-7 cells overexpressing GFP-PDLIM2 were seeded on coverslips and cultured to confluency in complete growth medium (DMEM and 10% FBS) for 48 h. Cells were fixed and nuclei were stained with Hoechst dye (blue). Cells were photographed at the focus plane of cell adhesion to the coverslip to demonstrate the location of GFP-PDLIM2 (green). Nuclei are shown in slightly different focus plane in the background. Note: nuclei appear to be larger or overlapping compared with GFP-PDLIM2 expression between the cells because GFP-PDLIM2 outlines the area of the cell that is adhered to the coverslip, which in a confluent monolayer, adopts to different shapes and sizes that do not represent the full body of the cell. **(B)** Suppression of PDLIM2 causes increased expression of IGF-1R and the scaffolding protein, RACK1. Control MCF10A cells (shScramble) or MCF10A cells with PDLIM2 expression stably suppressed (shPDLIM2) were cultured in a 3D Matrigel assay for 12 days. Cell structures were fixed and processed for confocal microscopy analysis for RACK1 (green) and IGF-1R (red) expression and nuclei were stained with Hoechst (blue), as described in Ref. (67).

The ability of cancer cells to invade, undergo reversible EMT, and retain stemness requires integrating gene expression with cytoskeleton dynamics and cell shape in response to cues from the extracellular matrix. PDLIM2 is an IGF-1 regulated cytoskeleton and nuclear protein that is also located in cell–cell and cell-matrix adhesion complexes (62, 63) (Figure 2A). PDLIM2 is expressed in epithelial cells, may be repressed in cancer, and is also highly expressed in cancer cells that exhibit an EMT phenotype (62–67). The *pdlim2* gene is located on chromosome 8p21, a region that is disrupted in many cancers and associated with metastasis (68). PDLIM2 regulates protein stability and expression of the key EMT markers, E-cadherin and Snail (63). Moreover, PDLIM2 regulates STAT and NFκB transcription factors and cytoskeleton function in inflammatory leukocytes, and the beta catenin transcriptional output in epithelial cells (63, 69–71). Suppression of PDLIM2 in normal MCF10A myoepithelial cells in 3D matrigel cultures leads to cell transformation, which, interestingly, is accompanied by markedly increased expression of both IGF-1R and RACK1 (Figure 2B). In addition, loss of PDLIM2 inhibits cell polarization and causes up-regulation of β1 integrin expression, and subsequent hyper-activation of downstream signaling through the FAK-RhoA-cofilin axis that can be reversed by pharmacological inhibition of FAK or Rho Kinases (67). Suppression of PDLIM2 in invasive cancer cells (DU145, MDA-MB-231) causes increased E-cadherin expression and cell–cell contact, loss of directional migration, altered expression and activity of many transcription factors associated with tumorigenesis, and reversal of EMT (63).

Since PDLIM2 silencing impairs the formation of polarized acinar structures and also suppresses EMT and directional migration, it regulates both E-cadherin-mediated cell–cell adhesion and ECM-Integrin activated signaling. This is consistent with a function as a cytoskeleton to nucleus courier protein, integrating signals from sites of cell adhesion with the cytoskeleton to gene expression in the nucleus. The presence of PDLIM2 in both cell–cell adhesion and cell–matrix adhesion complexes also suggests a role in signaling crosstalk. The most likely mechanisms for this are through controlling protein stability of key components of the adhesion complexes and the activity of their transcriptional regulators. PDLIM2 associates with the Cop9 signalosome [in particular, the CSN5 subunit (JAB1)], which regulates the activity of Cullin-E3 ligase complexes and protein degradation (63). However, it is not yet clear whether PDLIM2 function in adhesion complexes contributes directly to protein stability in these complexes or is associated with its sequestration away from the nucleus to enhance stability of transcription factors. Identifying the key targets of PDLIM2 within adhesion complexes will be necessary to establish its precise function.

## Do IGF-1R and IR Function Differently in Adhesion Signaling Complexes?

The insulin receptor isoform A (IR-A) has been firmly established as an important contributor to cancer phenotype, in particular, by promoting the renewal and survival of cancer stem cells and mediating responses to environmental conditions including hyperglycemia [reviewed in Ref. (10, 72, 73)] and resistance to IGF-1R-targeted therapies (74). Since cancer stem cell growth may require switching on an EMT phenotype, it will be interesting to establish whether the IGF-1R and IR-A function differently in cooperation with adhesion signaling. This issue is complicated by the fact that IGF-1R is activated preferentially by IGF-1 and the IR-A by IGF-2, and ligand-induced internalization and trafficking of the receptors may be different in response to ligand stimulation (29, 75, 76). Another key difference may reside in signaling or its regulation by the C terminal tails of these receptors. This region of the receptor exhibits least (approximately 40%) homology. In particular, the 1248-SFYYS-1252 motif in the C terminal tail of the IGF-1R lacks tyrosines in the IR and has the amino acid sequence SFFHS. Substitution of the tyrosines Y1250/Y1251 with phenylalanine in the IGF-1R is sufficient to impair recruitment of the IGF-1R into a complex with β1 integrin, and disrupt cooperative signaling and cytoskeleton organization (2, 77). Substitution of serine S1248 with alanine in the IGF-1R impairs migration slightly but increases ligand-independent survival (78). Internalization and trafficking of these mutant receptors is also impaired, but it remains to be determined whether the actions of this motif of the C terminal tails can distinguish between IGF-1R and IR-A activity and whether they behave similarly in cancer stem cell renewal and in promoting cell invasiveness in EMT. IGF-1R signaling adaptation may also be associated with DNA damage-directed therapy. Initial resistance to cisplatin in ovarian cancer has been associated with increased IGF-1R expression, whereas IGF-1R expression levels decrease in later stages of resistance (79). This again indicates an adaptation that involves suppression of IGF-1R expression levels, but not necessarily activity. Taken together, it is clear that IGF-1R expression is highly adaptable during cancer progression. It can cooperate with many other signaling pathways and may be influenced by different cellular responses and phenotypes. Thus, it may be a major contributor to cancer cell escape, survival, and ability to activate redundant cellular signaling pathways.

## Summary

IGF-1R signaling at focal adhesion complexes and its interplay with adhesion/cytoskeleton signaling has a critical role in cellular transformation, EMT, therapy resistance, and the plasticity of cancer cells. In addition to altered kinetics of canonical Akt and Erk signaling pathways, it is intimately involved in complex bi-directional cytoskeletal–nuclear signaling to determine gene expression necessary for cell polarity and phenotypic changes. Determining the key regulators of IGF-1R expression and how their expression is regulated in phenotypically distinct cancers may unlock new ways to target invasiveness and resistance to kinase inhibitors and conventional cancer therapies.

## Author Contributions

OC and RC prepared the manuscript. OC, SS, ET, MG, and RD reviewed manuscript and contributed to the design, execution, and interpretation of experiments for data, either published or unpublished, referred to in the manuscript.

## Conflict of Interest Statement

The authors declare that the research was conducted in the absence of any commercial or financial relationships that could be construed as a potential conflict of interest.

## Abbreviations

ECM, extracellular matrix; EMT, epithelial to mesenchymal transition; IGF-1R, insulin-like growth factor-1 receptor; IRS-1(2), insulin receptor substrate 1(2); NFκB, nuclear factor κB; PP2A, protein phosphatase 2A; STAT, signal transducer and activator of transcription.
